# Supplementary material for: Identification of photoperiod-induced specific miRNAs in the adrenal glands of Sunite sheep (Ovis aries)
Source: Front Vet Sci. 2022 Jul 22;9:888207. doi: 10.3389/fvets.2022.888207 (PMC9354845; doi:10.3389/fvets.2022.888207)
Supplement: Supplementary file 1 [file Data_Sheet_1.ZIP › Supplementary file/miRDeep2 file.zip!/miRDeep2 file/Know_Structure/SPLP42A1/oar-mir-369.pdf]

miRBase precursor : oar-mir-369
Total read count : 14744
oar-miR-369-5p read count : 1202
oar-miR-369-3p read count : 13538
remaining reads : 4

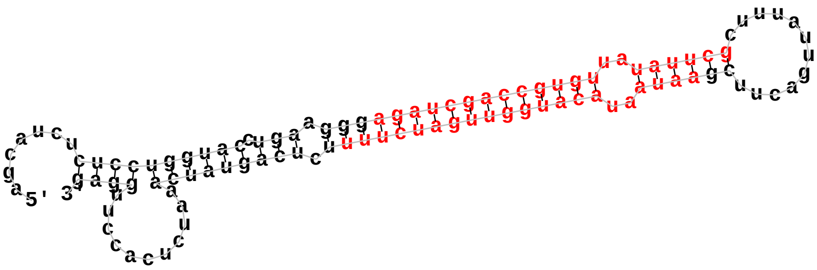

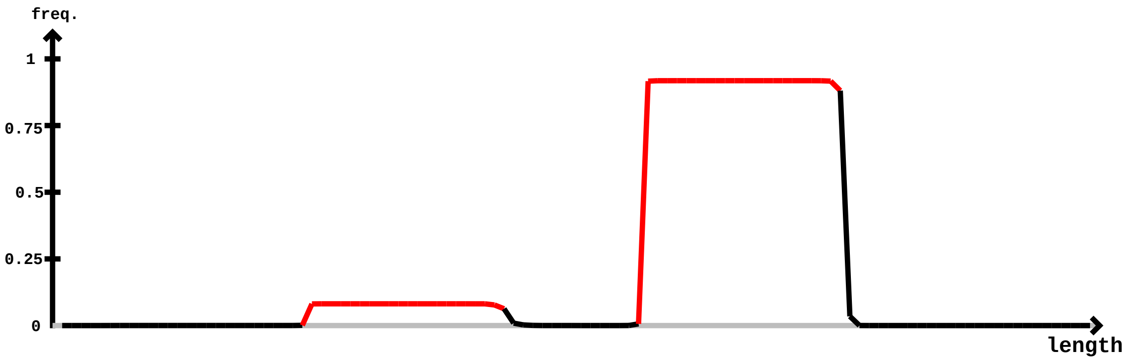

oar-miR-369-5p

oar-miR-369-3p

```
5'- agcaucucuccugguaccugaagggagaucgaccguguuauauucgcuuuauugacuucgaauaauacaugguugaucuuuucucaguaucaaaucucaccuuggag  -3'  exp
    .......((((((((((.(((.((((((((((((((((..((((((............))))))..)))))))))))))))).))))))))).........))))  reads  mm  sample
    ....ucucuccugguaccugaaggg..................................................................................  2  0  seq
    .....cucuccugguaccugaaggg..................................................................................  1  0  seq
    ........................gagaucgaccguguuauauu...............................................................  1  0  seq
    ........................gagaucgaccguguuauauucg.............................................................  9  0  seq
    .........................agaucgaccguguuaua.................................................................  1  0  seq
    .........................agaucgaccguguuauauu...............................................................  61  0  seq
    .........................agaucgaccguguuauauuc..............................................................  209  0  seq
    .........................agaucgaccguguuauauucg.............................................................  782  0  seq
    .........................agaucgaccguguuauauucgc............................................................  97  0  seq
    .........................agaucgaccguguuauauucgcu...........................................................  28  0  seq
    .........................agaucgaccguguuauauucgcuu..........................................................  8  0  seq
    .........................agaucgaccguguuauauucgcuuuauu......................................................  1  0  seq
    ..........................gaucgaccguguuauauu...............................................................  1  0  seq
    ..........................gaucgaccguguuauauuc..............................................................  1  0  seq
    ..........................gaucgaccguguuauauucg.............................................................  4  0  seq
    ..........................................................cgaauaauacaugguugaucuu...........................  1  0  seq
    ..........................................................cgaauaauacaugguugaucuuu..........................  3  0  seq
    ...........................................................gaauaauacaugguugaucu............................  3  0  seq
    ...........................................................gaauaauacaugguugaucuu...........................  8  0  seq
    ...........................................................gaauaauacaugguugaucuuu..........................  78  0  seq
    ...........................................................gaauaauacaugguugaucuuuu.........................  1  0  seq
    ............................................................aauaauacaugguugauc.............................  3  0  seq
    ............................................................aauaauacaugguugaucu............................  18  0  seq
    ............................................................aauaauacaugguugaucuu...........................  519  0  seq
    ............................................................aauaauacaugguugaucuuu..........................  12380  0  seq
    ............................................................aauaauaAaugguugaucuuu..........................  1  1  seq
    ............................................................aauaauacaugguugaucuuuu.........................  494  0  seq
    ............................................................aauaauacaugguugaucuuuuc........................  3  0  seq
    .............................................................auaauacaugguugaucuuu..........................  13  0  seq
    .............................................................auaauacaugguugaucuuuu.........................  11  0  seq
    ..............................................................uaauacaugguugaucuuuu.........................  1  0  seq
    ...............................................................aauacaugguugaucuuu..........................  1  0  seq
```
